# Transcriptome analysis reveals that fertilization with cryopreserved sperm downregulates genes relevant for early embryo development in the horse

**DOI:** 10.1371/journal.pone.0213420

**Published:** 2019-06-25

**Authors:** José M. Ortiz-Rodriguez, Cristina Ortega-Ferrusola, María C. Gil, Francisco E. Martín-Cano, Gemma Gaitskell-Phillips, Heriberto Rodríguez-Martínez, Katrin Hinrichs, Alberto Álvarez-Barrientos, Ángel Román, Fernando J. Peña

**Affiliations:** 1 Laboratory of Equine Reproduction and Equine Spermatology, Veterinary Teaching Hospital, University of Extremadura, Cáceres, Spain; 2 Department of Clinical and Experimental Medicine, Faculty of Medicine and Health Sciences, Linköping University, Linköping, Sweden; 3 Department of Veterinary Physiology and Pharmacology, College of Veterinary Medicine & Biomedical Sciences, Texas A&M University, College Station, Texas; 4 STAB, University of Extremadura, Badajoz, Spain; 5 Department of Biochemistry and Molecular Biology, University of Extremadura, Badajoz, Spain; Justus Liebig Universitat Giessen, GERMANY

## Abstract

Artificial insemination with cryopreserved spermatozoa is a major assisted reproductive technology in many species. In horses, as in humans, insemination with cryopreserved sperm is associated with lower pregnancy rates than those for fresh sperm, however, direct effects of sperm cryopreservation on the development of resulting embryos are largely unexplored. The aim of this study was to investigate differences in gene expression between embryos resulting from fertilization with fresh or cryopreserved sperm. Embryos were obtained at 8, 10 or 12 days after ovulation from mares inseminated post-ovulation on successive cycles with either fresh sperm or frozen-thawed sperm from the same stallion, providing matched embryo pairs at each day. RNA was isolated from two matched pairs (4 embryos) for each day, and cDNA libraries were built and sequenced. Significant differences in transcripts per kilobase million (TPM) were determined using (i) genes for which the expression difference between treatments was higher than 99% of that in the random case (P < 0.01), and (ii) genes for which the fold change was ≥ 2, to avoid expression bias in selection of the candidate genes. Molecular pathways were explored using the DAVID webserver, followed by network analyses using STRING, with a threshold of 0.700 for positive interactions. The transcriptional profile of embryos obtained with frozen-thawed sperm differed significantly from that for embryos derived from fresh sperm on all days, showing significant down-regulation of genes involved in biological pathways related to oxidative phosphorylation, DNA binding, DNA replication, and immune response. Many genes with reduced expression were orthologs of genes known to be embryonic lethal in mice. This study, for the first time, provides evidence of altered transcription in embryos resulting from fertilization with cryopreserved spermatozoa in any species. As sperm cryopreservation is commonly used in many species, including human, the effect of this intervention on expression of developmentally important genes in resulting embryos warrants attention.

## Introduction

Cryopreservation is a common procedure in assisted reproductive technology, in both humans and the animal breeding industry [1–3]. Cryopreserved sperm are routinely used for artificial insemination (AI), in vitro fertilization (IVF) and intracytoplasmic sperm injection (ICSI). However, it is clear that sperm cryopreservation methods are currently sub-optimal, as pregnancy rates with cryopreserved sperm are lower than those with fresh sperm in humans and horses [4–6], among other species. Cryopreservation leads to extensive damage of sperm cell membranes and causes metabolic and functional alteration of sperm [7, 8], particularly of their mitochondria [9–11]. Cryopreservation may alter sperm DNA [12]; recently, specific cryodamage to sperm genes and transcripts have been reported [13, 14], even in samples with good sperm motility post thaw and in the absence of detectable DNA fragmentation. The sperm DNA is epigenetically programmed to regulate embryonic gene expression, and changes to this epigenome cause developmental disregulation [15]. Cryopreservation has been found to significantly change the sperm DNA methylome, as well as to alter expression of epigenetic-related genes such as methyltransferases [12, 16]. Cryopreservation of sperm imposes oxidative stress and redox deregulation in spermatozoa, leading to the presence of toxic adduct-forming compounds such as 4- hydroxynonenal (4-HNE) in sperm membranes [17]. Moreover, mitochondria of spermatozoa surviving cryopreservation show increased production of reactive oxygen species [9, 10, 18]. Signaling pathways crucial to normal embryo development are sensitive to perturbations of endogenous redox state, and are also susceptible to modulation by reactive oxygen species [19]. Thus, fertilization by damaged spermatozoa may impact early embryo development and even have effects that appear later in the life of the offspring [20].

Moreover, appreciation of the contribution of sperm to embryo development has evolved from the concept that the only role of sperm at fertilization is to introduce the male genome into the egg. Sperm carry a myriad of small noncoding RNAs with potential roles in early embryo development [21, 22]. Notably, sperm carry the activating factor PLCζ, which triggers calcium oscillations that induce oocyte activation [23, 24], and it has been shown in mouse and rabbit that alterations in frequency and amplitude of post-fertilization calcium oscillations can affect the phenotype of the resulting embryo into post-implantation development and adulthood [25, 26]. Thus, there are extensive pathways by which cryopreservation of sperm could alter the development of the fertilized oocyte and embryo.

Despite the widespread use of cryopreserved sperm, and the known decrease in pregnancy rates with its use, little direct information is available on the effect of sperm cryopreservation on development of the resulting embryo. Recent advances in transcriptome amplification and next-generation sequencing provide the ability to obtain the full transcriptome of individual embryos [27], thus offering a basis for studies on differences in gene expression associated with fertilization with cryopreserved sperm. In the present study, we analyzed the transcriptome of equine embryos produced with fresh or frozen-thawed sperm, to determine the impact of sperm cryopreservation on gene expression during early equine embryo development.

## Material and methods

### Animals and experimental design

Animals belonging to and housed in our institution were maintained according to European laws and regulations, and all experimental procedures were reviewed and approved by the Ethical committee of the University of Extremadura, Cáceres, Spain. Six mares were used for this study; they were inseminated with the same stallion of known fertility to reduce genetic variability [28]. Each mare was assigned a day of embryo recovery (8, 10 or 12 days post ovulation) and on successive cycles was assigned to be inseminated with fresh or frozen-thawed sperm from the same stallion, to provide a matched embryo pair for that day of embryo development. The mares were treated with a prostaglandin analogue to shorten the luteal phase and were monitored daily by transrectal ultrasonography. When a follicle of at least 35 mm diameter was detected in the absence of luteal tissue, with marked uterine edema and low cervical tone, mares received 2,500 IU of hCG i.v.. The follicle was monitored by transrectal ultrasonography every 6 h thereafter to detect the time of ovulation. Mares were inseminated immediately once ovulation was detected, with a minimum of 100 million either fresh sperm or frozen-thawed sperm, from the same stallion and ejaculate. For this, semen was collected, and half of the ejaculate was processed as fresh semen for the immediate insemination of the mare. The other half was frozen following the standard protocol in our center [17, 29, 30], and stored in LN for the next insemination of the same mare. Following this protocol, each mare was inseminated with the same ejaculate, first with the fresh extended aliquot and on a second cycle with the frozen-thawed aliquot.

Embryos were obtained by uterine lavage on the designated day after ovulation. For each embryo day, 2 embryos produced with fresh sperm, designated FRSH embryos, and 2 embryos produced with frozen-thawed sperm, designated CRYO embryos were obtained. Embryos were snap-frozen in liquid N_2_ and stored at -80°C until analysis. Previous clinical reports indicated that there is no a significant effect in the rate of embryonic vesicle growth between mares inseminated with fresh or frozen-thawed sperm if both are inseminated post-ovulation [31].

### Isolation of RNA

Total RNA was isolated from the embryos using the PicoPure RNA Isolation Kit (Catalog number KIT0204, Thermofisher) following the manufacturer’s instructions. RNA concentration and quality were assessed by automatic electrophoresis using 2100 Bioanalyzer (Agilent Technologies, Santa Clara, CA, USA).

### RNA-seq analysis

cDNA libraries were built using an IonTorrent S5/XL sequencer (Thermo Fisher Scientific, Waltham, MA USA). The raw reads were aligned to a horse transcriptome generated using ENSEMBL (Equ Cab 2 version) in the Torrent server with proprietary ThermoFisher algorithms. Then, custom scripts were used to transform reads into transcript counts, and transcripts per kilobase million (TPM) scores for each gene were retrieved. A gene was considered expressed if the reads per kilobase or transcript model per million mapped reads was > 0.4. In order to evaluate gene expression differences between treatments (FRSH or CRYO embryos), we calculated two thresholds: first, we calculated the random TPM differences between FRSH and CRYO embryos by permutation of the TPM gene scores. Then we chose the genes whose expression difference between the two conditions was higher than in 95% (P<0.05) or in 99% (P<0.01) of the random cases. As a second score, we used a fold change ≥ 2 as a threshold in order to avoid expression biases in the selection of the candidate genes.

### Gene ontology and pathway analysis

The annotations of the candidate genes selected after the RNA-seq analyses were explored to detect significant differences in molecular pathways between treatments. Specifically, the DAVID webserver [32] was used to retrieve the terms (gene ontology, up-expressed tissues, KEGG and reactome pathways, protein-protein interactions, etc.) with significant over-presence of the candidate genes, using a false discovery rate (FDR) < 0.05. We used the human genome as reference for the analysis because of its increased depth in terms of annotation.

### Network analysis

STRING [33] was used to analyze the internal structure of the functional network obtained using the candidate genes. Data included co-expression, genetic fusion, co-occurrence or protein-protein interactions, among others. A high threshold (0.700) was selected for positive interaction between a pair of genes.

## Results

A total of 12 conceptuses were analysed (2 FRSH and 2 CRYO at each day). An average of 29,196 transcripts per embryo were obtained.

### Day-8 embryos

**Fig 1:**
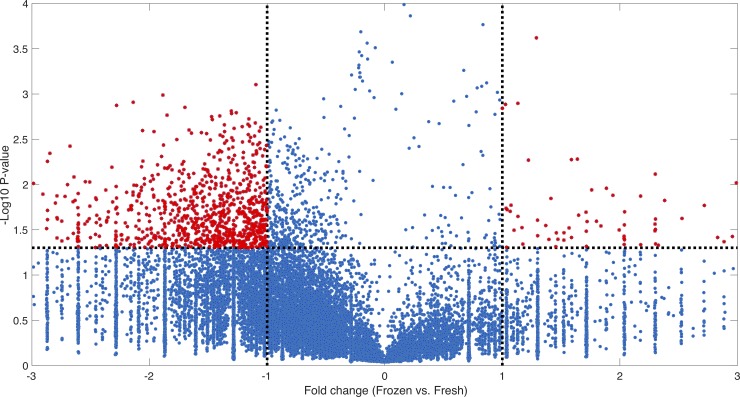
Volcano plot representing the RNA-seq results for Day-8 equine embryos conceived using fresh sperm (FRSH) or frozen-thawed sperm (CRYO). Each point represents a gene. On the X-axis, the fold change was calculated as the log2-ratio between the average gene expression in CRYO embryos and the average gene expression in FRSH embryos. Therefore, positive values indicate genes in which expression is higher in CRYO embryos, while negative values indicate genes whose expression is higher in FRSH embryos. On the Y-axis, the statistical significance for the difference in gene expression (see Methods) is represented as the (-log10) of the P-value. Dashed lines indicate the thresholds for significance on the two axes (-1 and +1 in the case of the X-axis for up-regulated and down-regulated genes, respectively, in CRYO embryos; and 1.30 (equal to a P-value = 0.05) in the case of the Y-axis). Red points mark differentially expressed genes.

In Day-8 CRYO embryos, 100 transcripts showed increased abundance and 157 transcripts showed decreased abundance in respect to FRSH embryos of the same age from the same stallion and mare (Fig 1).

**Fig 2:**
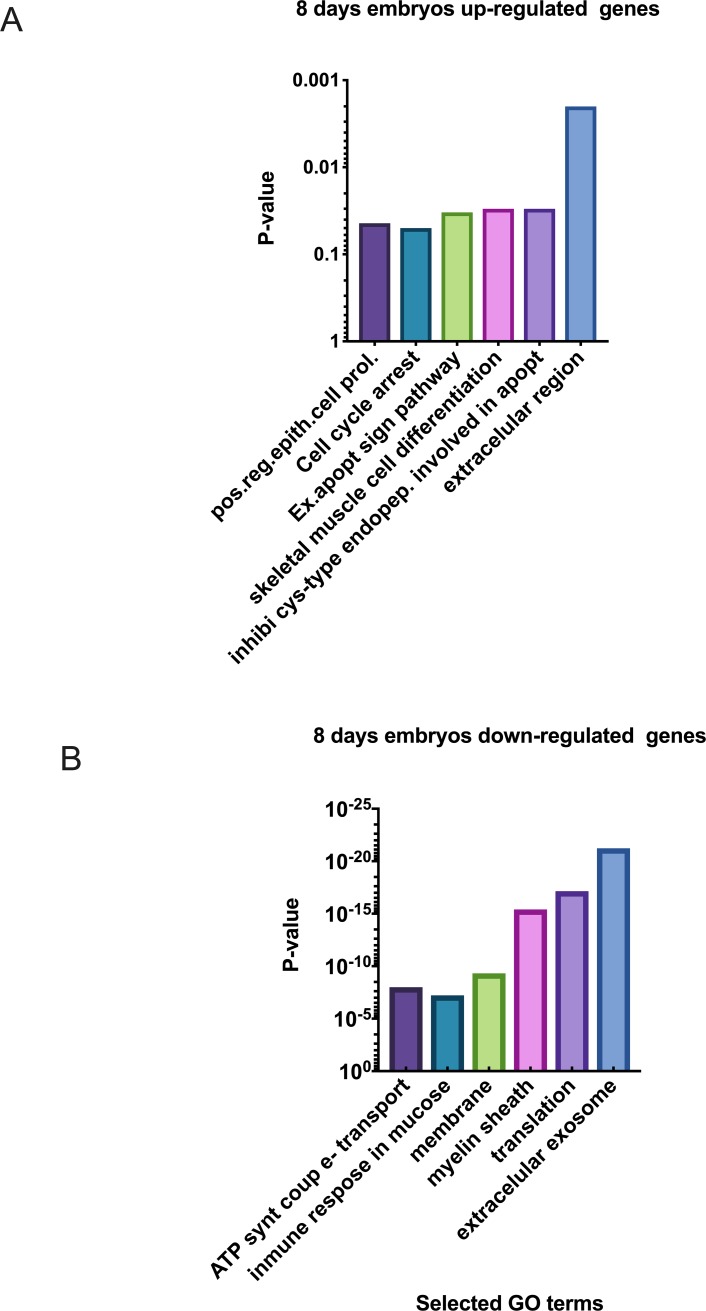
Selected enriched GO terms differentially regulated in Day-8 equine embryos obtained using fresh sperm (FRSH) and frozen-thawed sperm (CRYO), (A) transcripts down regulated in 8-Day CRYO embryos, (B) transcripts up regulated in 8-Day CRYO embryos.

Of the 100 transcripts showing increased abundance in CRYO embryos, 23 could be aligned to the genome build (S1 Table). These included the progesterone receptor membrane component (8PGRMC1). Enriched biological processes (Fig 2A) included extracellular region genes, defensing beta 119, insulin like 3, prostaglandin D2 synthase and uteroglobin; genes associated with negative regulation of cysteine type endopeptidase activity involved in apoptotic processes including nuclear receptor subfamily 4 group A member and paired box 2; and genes involved in skeletal muscle cell differentiation including activating transcription factor 3 and nuclear receptor subfamily 4 group 4 A member. STRING analysis revealed no significant enrichments in functional networks for transcripts with increased abundance.

**Fig 3:**
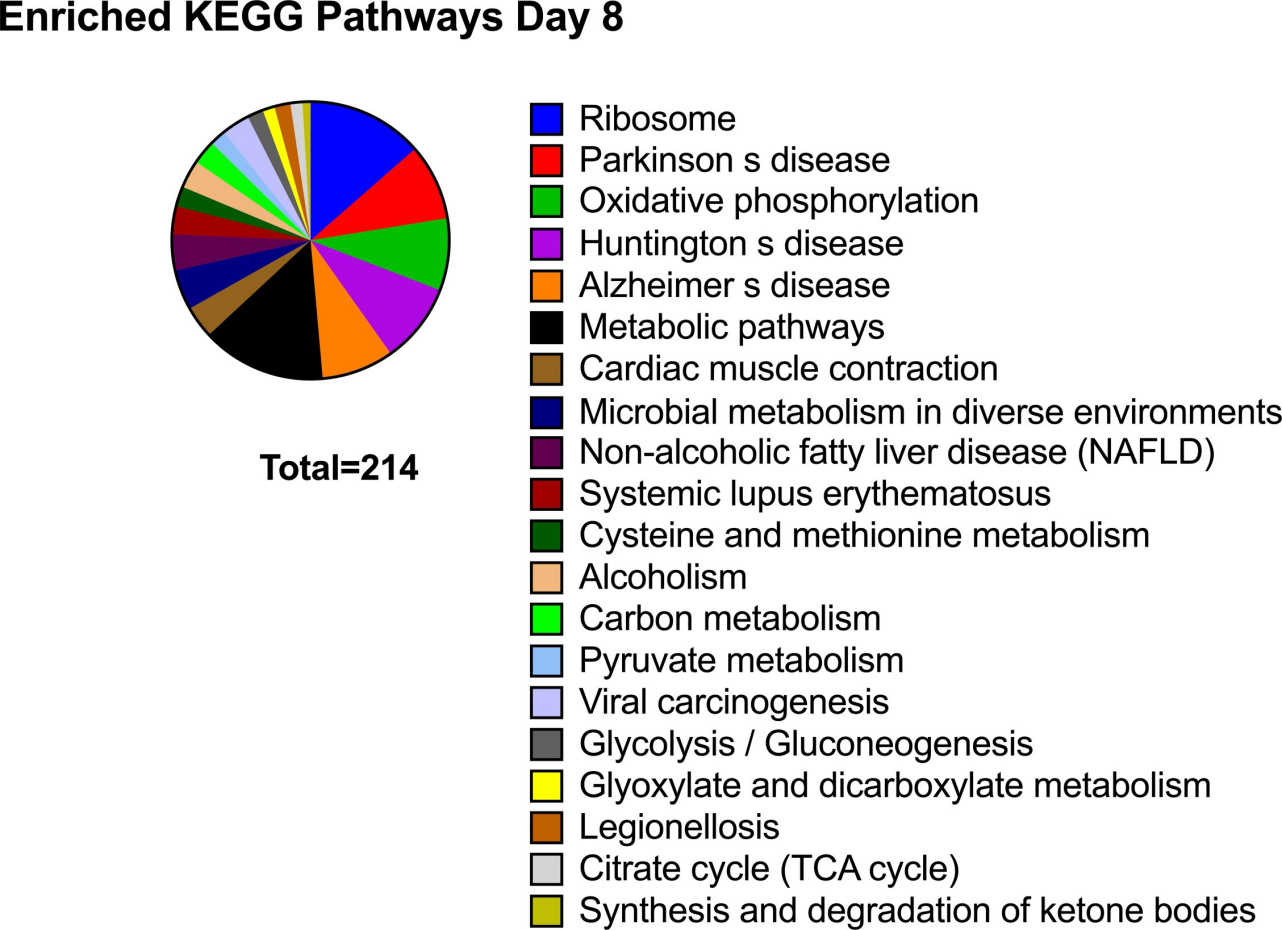
Enriched Kyoto Encyclopedia of Genes and Genomes (KEGG) pathways in transcripts downregulated in 8-Day embryos obtained with frozen-thawed spermatozoa.

**Fig 4:**
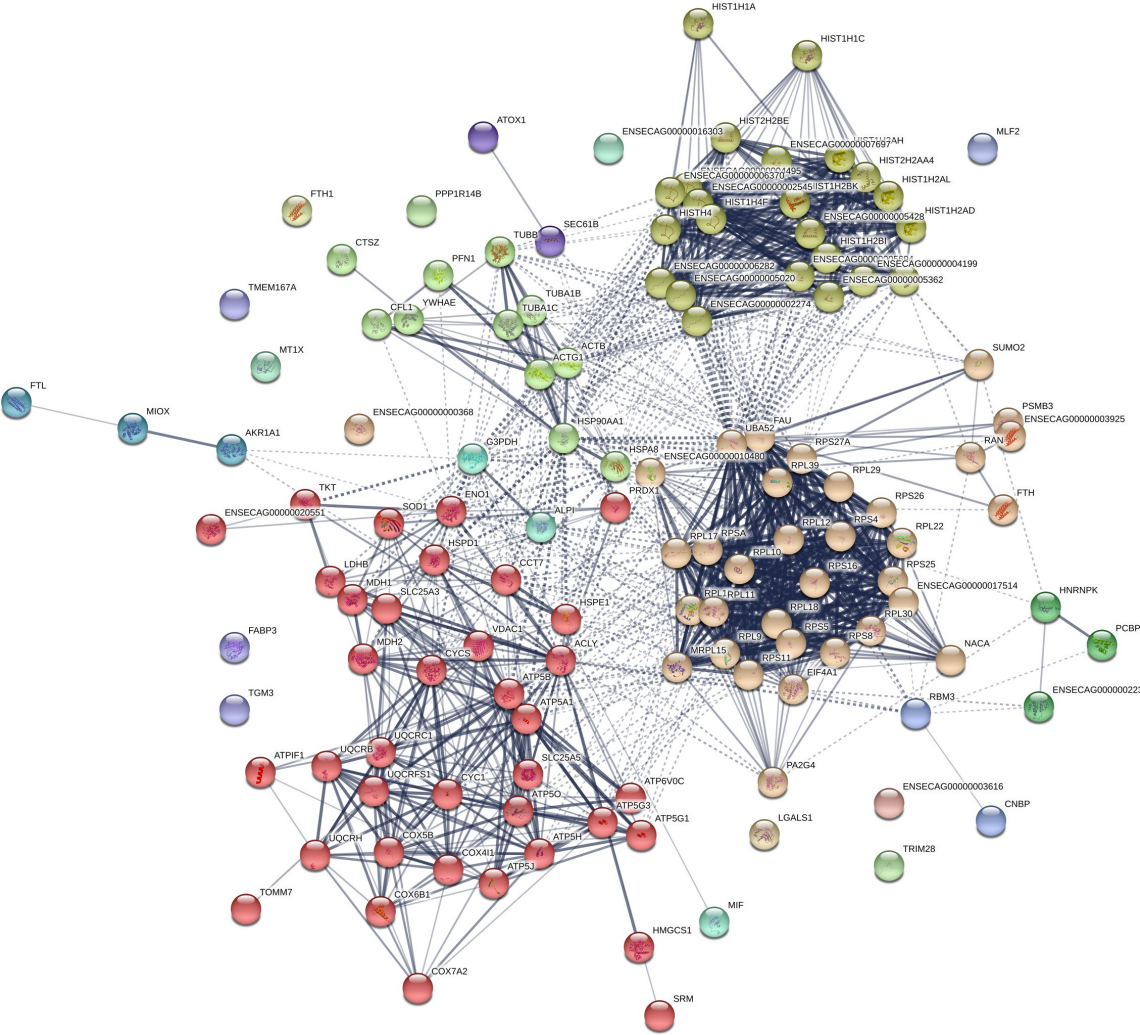
Functional networks (STRING) of transcripts downregulated in 8-Day equine embryos obtained with frozen-thawed sperm (CRYO embryos). Functional networks apply to histones and mitochondrial proteins. Controls are 8-Day embryos from the same mare, obtained with fresh semen from the same ejaculate that was frozen and used to produce the CRYO embryos. A list of the transcripts in each cluster obtained after STRING analysis are presented. Colors for each cluster are given in S3 Table.

Transcripts showing decreased abundance in CRYO embryos provided more information, with 129 transcripts annotated in the equine database. The complete list of transcripts is presented in S2 Table. Due to the large number of genes retrieved, the threshold was reset at P< 0.001 and 62 transcripts were then retrieved (Table 1). Related gene ontology terms are shown in Fig 2B. Enriched terms in KEGG (Kyoto encyclopedia of gene and genomes) pathways included ribosome, Parkinson disease and oxidative phosphorylation (Fig 3). STRING analysis, performed using a threshold of 0.700, obtained a protein-protein interaction (PPI) enrichment P value of < 1.0 x10^-16^ (Fig 4). The complete list of genes in this network with their clustering is presented in S3 Table. Enriched biological processes included cellular process, iron ion transport, cellular iron ion homeostasis, metabolic process, response to inorganic substance, biological regulation, single-organism process, cellular macromolecule metabolic process, single organism cellular process, cellular metabolic process, response to stimulus, cellular response to zinc ion, transport, regulation of biological process, oxidation-reduction process, cellular component disassembly, cellular nitrogen compound metabolic process, translation, single organism transport, gene expression, positive regulation of nitrogen compound metabolic process, biological process, protein folding, cellular component organization, regulation of cell proliferation, and primary metabolic process.

**Table 1:** Enriched biological processes from DEGs (downregulated) in 8 days embryos obtained after AI with frozen thawed sperm, as identified by DAVID functional annotation analysis.

Functional terms of overrepresented biological processes ^a^	P value ^b^
Chromosome (21, 35.78)	7.1 x10^-26^
Nucleosome core (19, 42.08)	9.2 x10^-25^
Extracellular exosome (62, 3.56)	5.7 x10^-22^
Histone fold (19, 30.02)	7.5 x10^-22^
Structural constituent of ribosome (24, 13.82)	5.8 x10^-20^
Ribosome (24, 12.89)	1.21 x10^-19^
Histone core (15, 36.91)	1.69 x10^-18^
Translation (21, 14.65)	7.15 x10^-18^
Mylein sheath (18, 16.63)	3.87 x10^-16^
Nucleosome (13, 31.72)	4.20 x10^-15^
Ribonucleoprotein (14, 24.48)	1.15 x10^-14^
Ribosomal protein (13, 29.28)	1.39 x10^-14^
Nucleosome assembly (13, 23.18)	2.14 x10^-13^
Poly (A) RNA binding (34, 4.29)	4.69 x10^-13^
Nuclear nucleosome (10, 40.67)	1.37 x10^-12^
Parkinson’s disease (18, 9.52)	2.60 x10^-12^
Cytosolic small ribosomal subunit (10, 33.98)	8.65 x10^-12^
Cytosolic large ribosomal subunit (11, 24.85)	1.46 x10^-11^
Systemic lupus erythematosus (16, 10.14)	2.51 x10^-11^
Hungtinton’s disease (19, 7.38)	4.19 x10^-11^
H2B (8, 52.06)	7.18 x10^-11^
Nucleus (26, 4.79)	8.79 x10^-11^
Oxidative phosphorylation (16, 9.01)	1,43 x10^-10^
Histone H2B (8, 48.75)	1.48 x10^-10^
DNA binding (21, 6.06)	1.81 x10^-10^
Membrane (27, 4.21)	4.82 x10^-10^
Alzheimer disease (17, 7.38)	6.32 x10^-10^
Alcoholism (16, 7.17)	3.64 x10^-9^
ATP synthesis coupled proton transport (7, 41.39)	1.0x10^-8^
Innate immune response in mucose (6, 51.80)	5.89x10^-8^
Antibacterial humoral response (6, 48.15)	9.1x10^-8^
Focal adhesion (15, 6.10)	1.38x10^-7^
DNA binding (18, 4.18)	1.02x10^-6^
DNA replication dependent nucleosome assembly (6, 30.64)	1.13x10^-6^
Hydrogen ion transport (5, 55.38)	1.38x10^-6^
Protein heterotetramerizacion (6, 29.31)	1.44x10^-6^
Proton transporting ATP synthase activity, rotational mechanism (5, 51.26)	1.66x10^-6^
Cytoplasm (11, 5.91)	1.60x10^-5^
Cytoplasmatic translation (5, 29.56)	2.00x10^-5^
H4 (4, 60.63)	2.86 x10^-5^
Viral carcinogenesis (13, 4.39)	3.09x x10^-5^
Cardiac muscle contraction (8, 8.53)	3.57 x10^-5^
Histone H4 (4, 56.81)	3.68 x10^-5^
Histone H4 conserved site (4, 56.81)	3.68 x10^-5^
TAF (4, 56.88)	5.56 x10^-5^
Defense response to gram positive bacterium (6, 49.69)	6.14 x10^-5^
Tata Box binding protein associated factor (TAF) (4, 14.04)	7.14 x10^-5^
Negative regulation of megakaryocite differentiation (4, 46.53)	1.04 x10^-4^
ATP hydrolysis coupled ion transport (5, 40.85)	1.14 x10^-4^
Acetylation (6, 19.37)	1.32 x10^-4^
H2A (4, 12.08)	3.67 x10^-4^
Mitochondrial electron transport, cytochrome c to oxygen (3, 27.33)	4.57 x10^-4^
Ribosomal large subunit assembly (4, 84.27)	4.94 x10^-4^
DNA replication independent nucleosome assembly (4, 24.96)	4.94 x10^-4^
Histone H2A (4, 24.37)	5.44 x10^-4^
V-ATPase proteolipid subunit C-like domain (3, 76.78)	5.86 x10^-4^
DNA templated transcription, initiation (4, 22.47)	6.81 x10^-4^
Nuclear chromosome, telomeric region (6, 8.22)	7.79 x10^-4^
Non alcoholic fatty liver disease (NAFLD) (9, 4.40)	8.75 x10^-4^
Lactate/malate dehydrogenase (3, 63.99)	8.74 x10^-4^
Lactate malate dehydrogenase, N–terminal (3, 63.99)	8.74 x10^-4^
Mitochondrial proton transporting ATP synthase complex (3, 61.00)	9.60 x10^-4^

^a^ Values in parenthesis represent the number of genes involved in and the fold enrichment of the corresponding functional terms

^b^ EASE score examine the significance of gene term enrichment with a modified Fisher’s exact test

### Day-10 embryos

In Day-10 embryos 239 transcripts showed increased abundance (P < 0.01), and 206 showed decreased abundance, in CRYO embryos in comparison with FRSH embryos.

**Fig 5:**
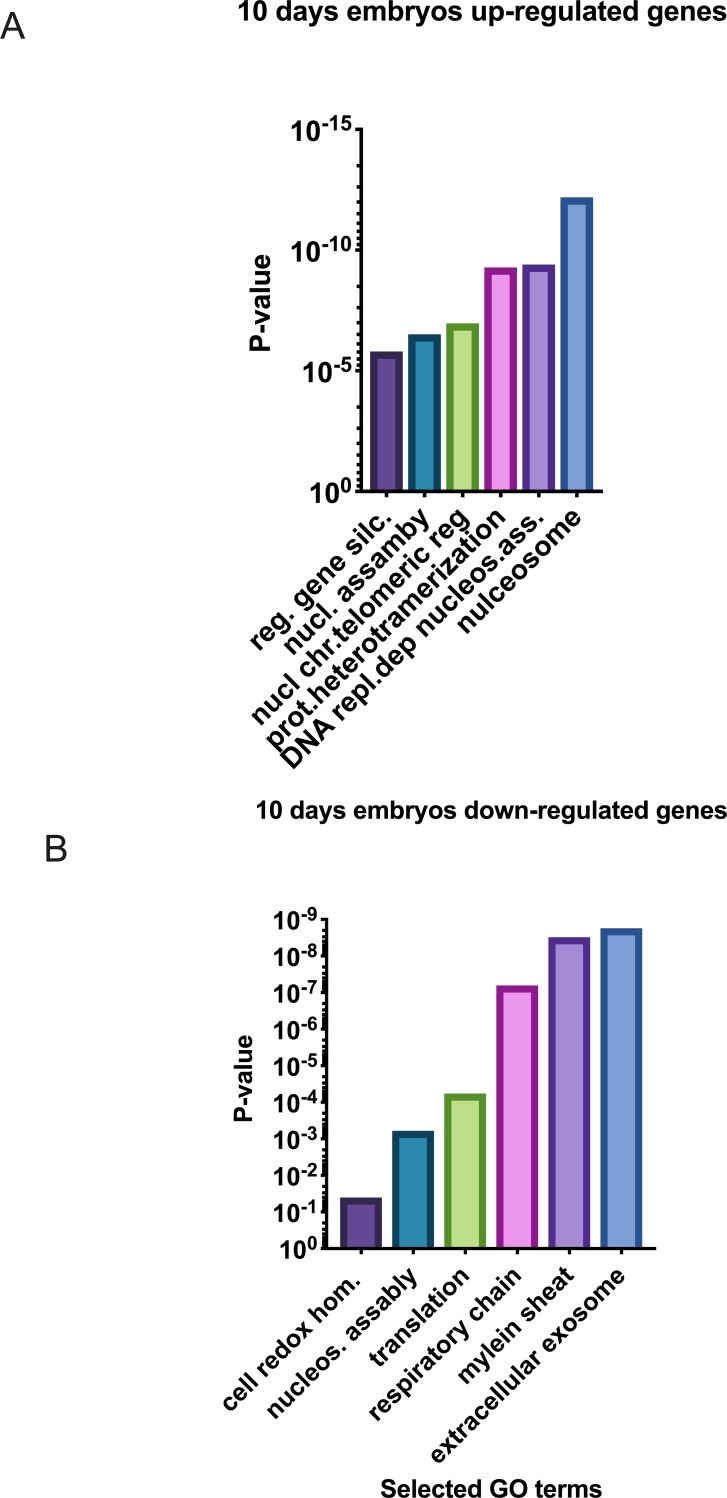
Selected enriched GO terms differentially regulated in 10-Day equine embryos obtained with fresh sperm (FRSH) and frozen-thawed sperm (CRYO), (A) transcripts down regulated transcripts 10-Day CRYO embryos, (B) transcripts up regulated in 10-Day CRYO embryos.

Of the 239 transcripts showing increased abundance in CRYO embryos, 53 aligned to the genome build (S4 Table). Functional annotation revealed these genes to be related to the GO terms and KEGG pathways nucleosome, systemic lupus erythematatosus, DNA replication-dependent nucleosome assembly, protein heterodemerization, alcoholism, nuclear chromosome, telomeric region, regulation of gene silencing, nucleosomal DNA binding, membrane, translation, poly (A) RNA binding, viral carcinogenesis, negative regulation of megakaryocyte differentiation, DNA replication independent nucleosome assembly, extracellular exosome, DNA-templated transcription, xenophagy, ribosome, positive regulation of defense to virus by host, DNA binding, mitochondrion, cytosolic large ribosomal subunit, extracellular space, transcriptional misregulation in cancer, innate immune response in mucosa, U1 snRNP, antibacterial humoral response, telomerase RNA binding and mitochondrial small ribosomal subunit (Fig 5A). STRING analysis revealed a PPI enrichment P value of < 1.0 x10^-16^. Functional enrichment included the PFAM protein domain Core histone H2A/H2B/H3/HA and the INTERPRO protein domains, including Histone fold, Histone H3/CNEP-A, Histone H2A/H2B/H3, Histone H4, Histone H4 conserved site, TATA box binding protein associated factor (TAF) and ribosomal protein L23/L15e core domain.

**Fig 6:**
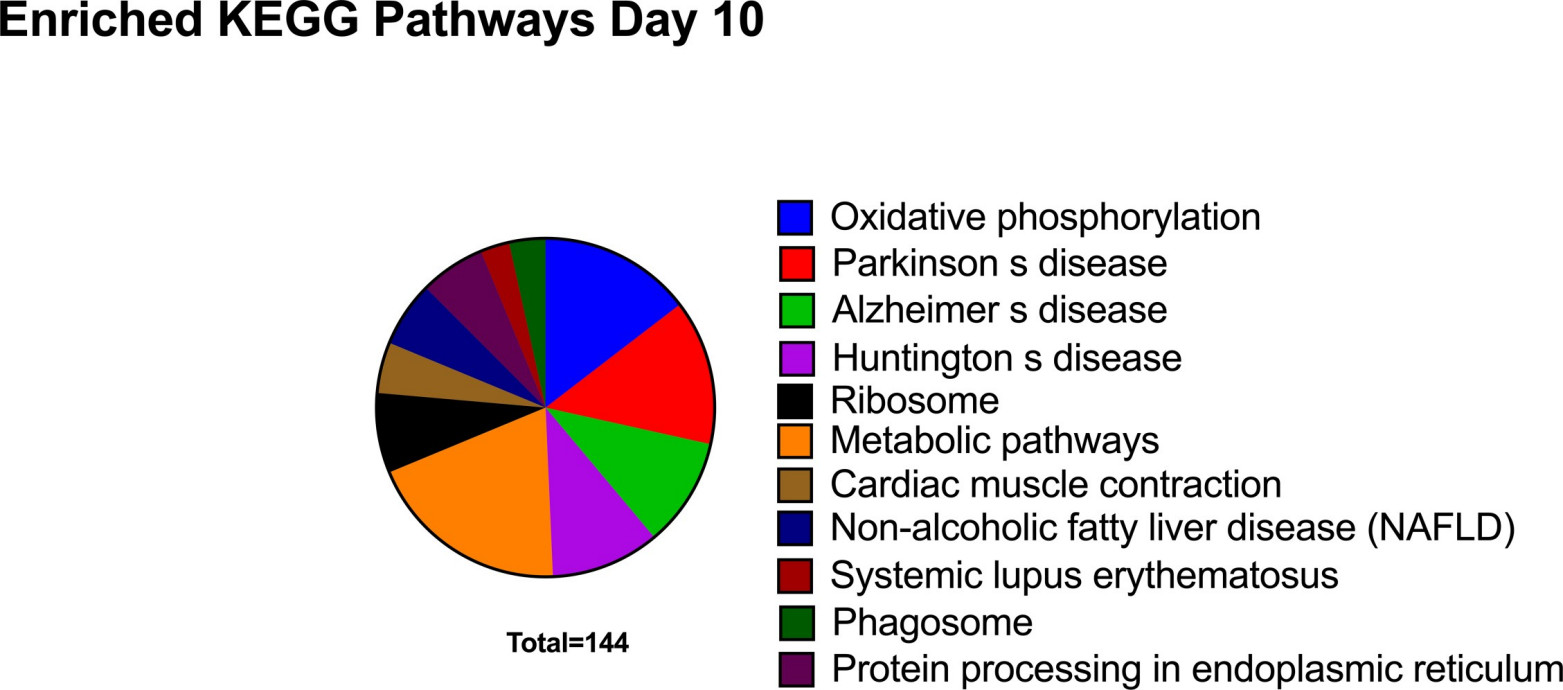
Enriched Kyoto Encyclopedia of Genes and Genomes (KEGG) pathways in transcripts downregulated in 10-Day embryos obtained with frozen-thawed spermatozoa.

**Fig 7:**
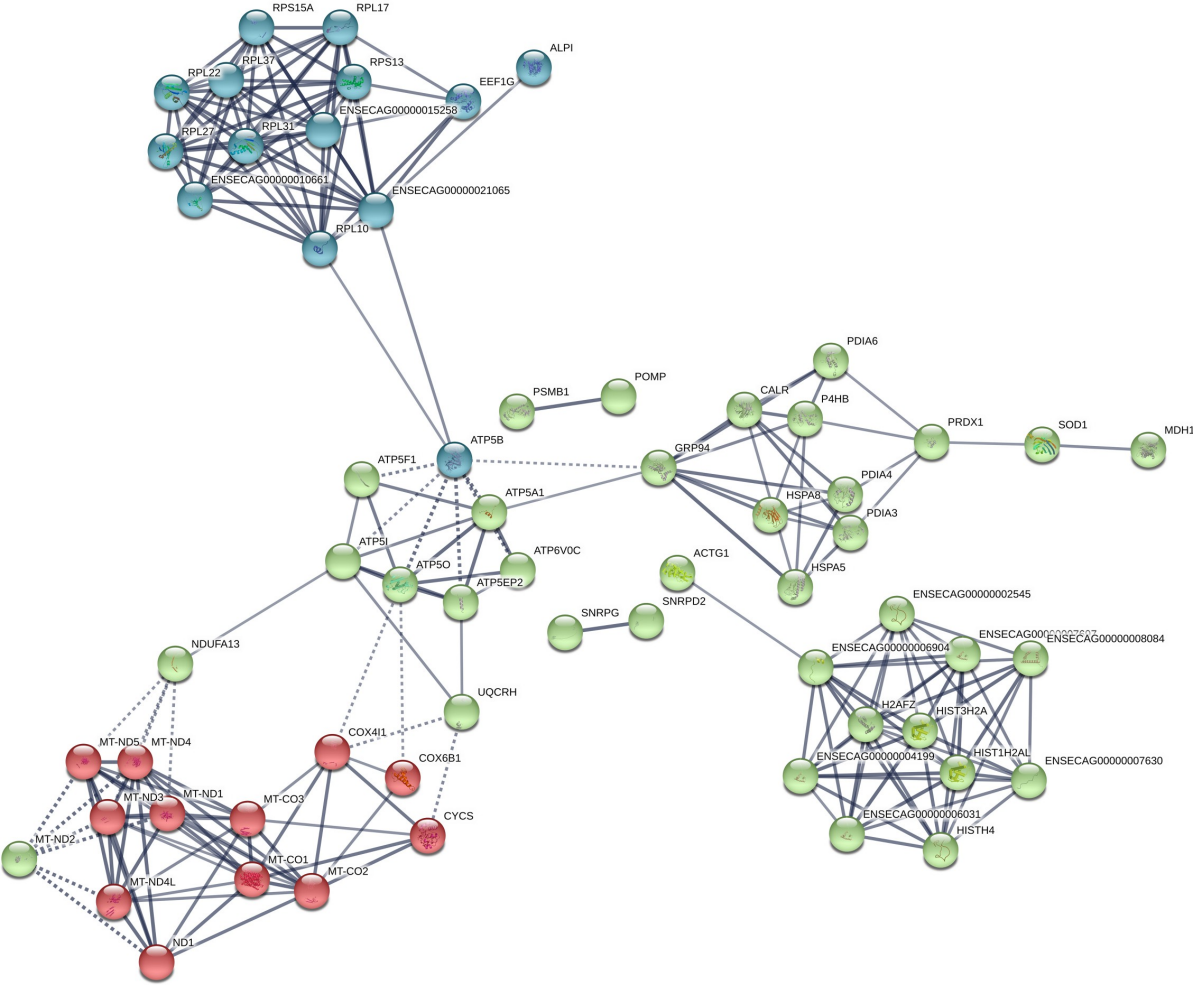
Functional networks (STRING) of transcripts downregulated in 10-Day equine embryos obtained with frozen-thawed sperm (CRYO embryos). Functional networks apply to histones and mitochondrial proteins. Controls are same-age embryos from the same mare, obtained with fresh semen from the same ejaculate that was frozen and used to produce the CRYO embryos.

Of the 206 transcripts showing decreased abundance in CRYO embryos at Day 10, 115 were aligned. Enriched KEGG pathways that were also detected in Day-8 embryos (Table 2) included oxidative phosphorylation, Parkinson disease, Alzheimer disease, Hungtington disease, Metabolic pathways, Ribosome, cardiac muscle contraction, and non-alcoholic fatty liver disease. Three new KEGG enriched pathways, protein processing in endoplasmic reticulum, systemic lupus erythematosus and phagosome, were detected (Fig 6). More significantly represented GO terms were ATP synthesis coupled proton transport, translation, nucleosome assembly, cell redox homeostasis, extracellular exosome, myelin sheath, respiratory chain, mitochondrion, extracellular space, NADH dehydrogenase (ubiquinone) activity, structural constituent of ribosome, and proton transporting ATP synthase activity rotational mechanism (Fig 5B). A complete list of enriched GO terms retrieved are given in Table 3. STRING analysis revealed functional networks with a PPI enrichment P value of < 1.0 x10^-16^ (Fig 7). Functional enrichment included the PAFM domains core histone H2A/H2B/H3/H4, thiorredoxin, NADH deshidrogenase, NADH-Ubiquinone and plastoquinone (Complex I), various chains.

**Table 2:** Selected enriched Kyoto Encyclopedia of Genes and Genomes (KEGG) pathways enriched in downregulated transcripts of in 10 days embryos obtained after AI with frozen thawed sperm.

KEGG pathway	Pathway description	observed gene count	false discovery rate
190	Oxidative phosphorylation	21	2,45E-23
5012	Parkinson s disease	20	4,64E-21
5010	Alzheimer s disease	15	1,05E-12
5016	Huntington s disease	15	3,42E-12
1100	Metabolic pathways	28	4,29E-09
3010	Ribosome	11	8,96E-09
4260	Cardiac muscle contraction	7	2,56E-06
4932	Non-alcoholic fatty liver disease (NAFLD)	9	3,96E-06
4141	Protein processing in endoplasmic reticulum	9	1,61E-05

**Table 3:** Gene ontology annotations enriched in downregulated transcripts of 10 days embryos obtained after AI with frozen thawed sperm.

Term	P Value
GO:0070062~extracellular exosome	1,72E-09
GO:0043209~myelin sheath	3,03E-09
GO:0070469~respiratory chain	6,30E-08
GO:0022625~cytosolic large ribosomal subunit	5,33E-07
GO:0008137~NADH dehydrogenase (ubiquinone) activity	8,07E-07
GO:0005747~mitochondrial respiratory chain complex I	3,12E-06
GO:0015986~ATP synthesis coupled proton transport	4,78E-06
GO:0003735~structural constituent of ribosome	1,66E-05
GO:0000788~nuclear nucleosome	2,25E-05
GO:0046933~proton-transporting ATP synthase activity, rotational mechanism	2,90E-05
GO:0005925~focal adhesion	4,41E-05
GO:0006412~translation	5,75E-05
GO:0046961~proton-transporting ATPase activity, rotational mechanism	1,59E-04
GO:0000786~nucleosome	1,73E-04
GO:0042773~ATP synthesis coupled electron transport	2,22E-04
GO:0006334~nucleosome assembly	5,99E-04
GO:0005743~mitochondrial inner membrane	0,001576512
GO:0004129~cytochrome-c oxidase activity	0,002995144
GO:0005739~mitochondrion	0,00444262
GO:0006336~DNA replication-independent nucleosome assembly	0,005364717
GO:0045261~proton-transporting ATP synthase complex, catalytic core F(1)	0,011191606
GO:0015991~ATP hydrolysis coupled proton transport	0,013628447
GO:0006457~protein folding	0,017943471
GO:0051603~proteolysis involved in cellular protein catabolic process	0,019505148
GO:0003677~DNA binding	0,022056606
GO:0006123~mitochondrial electron transport, cytochrome c to oxygen	0,024396132
GO:0044822~poly(A) RNA binding	0,032707627
GO:0005753~mitochondrial proton-transporting ATP synthase complex	0,0332053
GO:0005615~extracellular space	0,040768468
GO:0005687~U4 snRNP	0,044030068
GO:0045454~cell redox homeostasis	0,047994139
GO:0006122~mitochondrial electron transport, ubiquinol to cytochrome c	0,048204941
GO:1902166~negative regulation of intrinsic apoptotic signaling pathway in response to DNA damage by p53 class mediator	0,054067016
GO:0006120~mitochondrial electron transport, NADH to ubiquinone	0,059893472
GO:0045653~negative regulation of megakaryocyte differentiation	0,065684522
GO:0030330~DNA damage response, signal transduction by p53 class mediator	0,065684522
GO:0016020~membrane	0,071097195
GO:0004185~serine-type carboxypeptidase activity	0,07400431
GO:0034719~SMN-Sm protein complex	0,075791838
GO:0005685~U1 snRNP	0,075791838
GO:0002227~innate immune response in mucosa	0,077161252
GO:0007569~cell aging	0,077161252
GO:0005682~U5 snRNP	0,080983251
GO:0071157~negative regulation of cell cycle arrest	0,082847353
GO:0019731~antibacterial humoral response	0,082847353
GO:0005975~carbohydrate metabolic process	0,083234703
GO:0005686~U2 snRNP	0,086145885
GO:0030970~retrograde protein transport, ER to cytosol	0,088498889
GO:0000784~nuclear chromosome, telomeric region	0,089088094
GO:0045787~positive regulation of cell cycle	0,094116067

### Day-12 embryos

**Fig 8:**
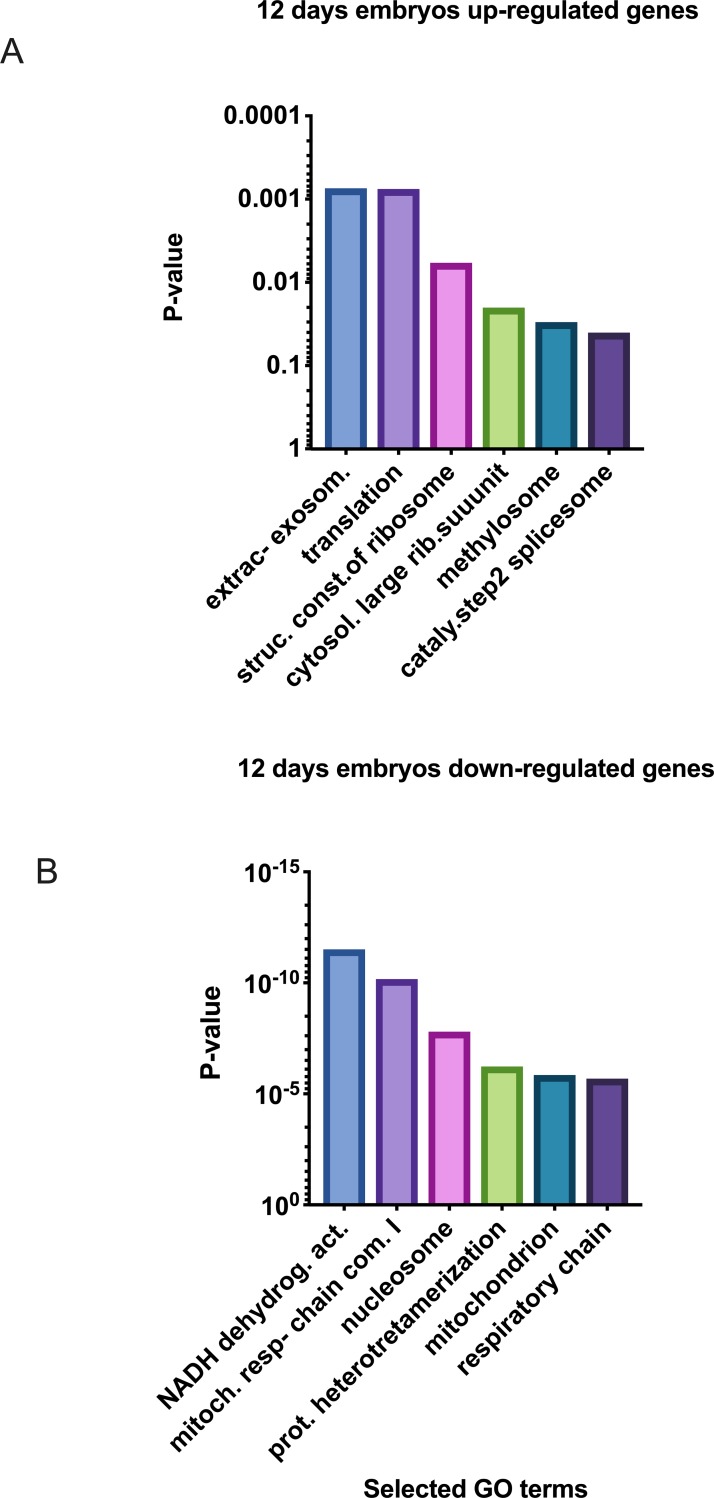
Selected enriched GO terms differentially regulated in 12-Day equine embryos obtained with fresh sperm (FRSH) and frozen-thawed sperm (CRYO), (A) transcripts down regulated in 12-Day CRYO embryos, (B) transcripts up regulated in 12-Day CRYO embryos.

In Day-12 embryos, 149 transcripts showed increased abundance and 157 showed decreased abundance in CRYO embryos. Of the 149 transcripts with increased abundance, 61 were annotated (S5 Table). Enriched KEGG pathways included ribosome and Parkinson disease and the GOterms extracellular exosome, translation, structural constituent of ribosome, nuclear nucleosome, mitochondrial respiratory chain complex I, cytosolic large ribosomal subunit, nucleosome assembly, methylosome, and catalytic step 2 spliceosome (Fig 8A). On STRING analysis, the KEGG pathways Ribosome (Pathway ID 03010) and Parkinson disease (Pathway ID 05012) showed a PPI enrichment P value of < 8 x10^-10^.

**Fig 9:**
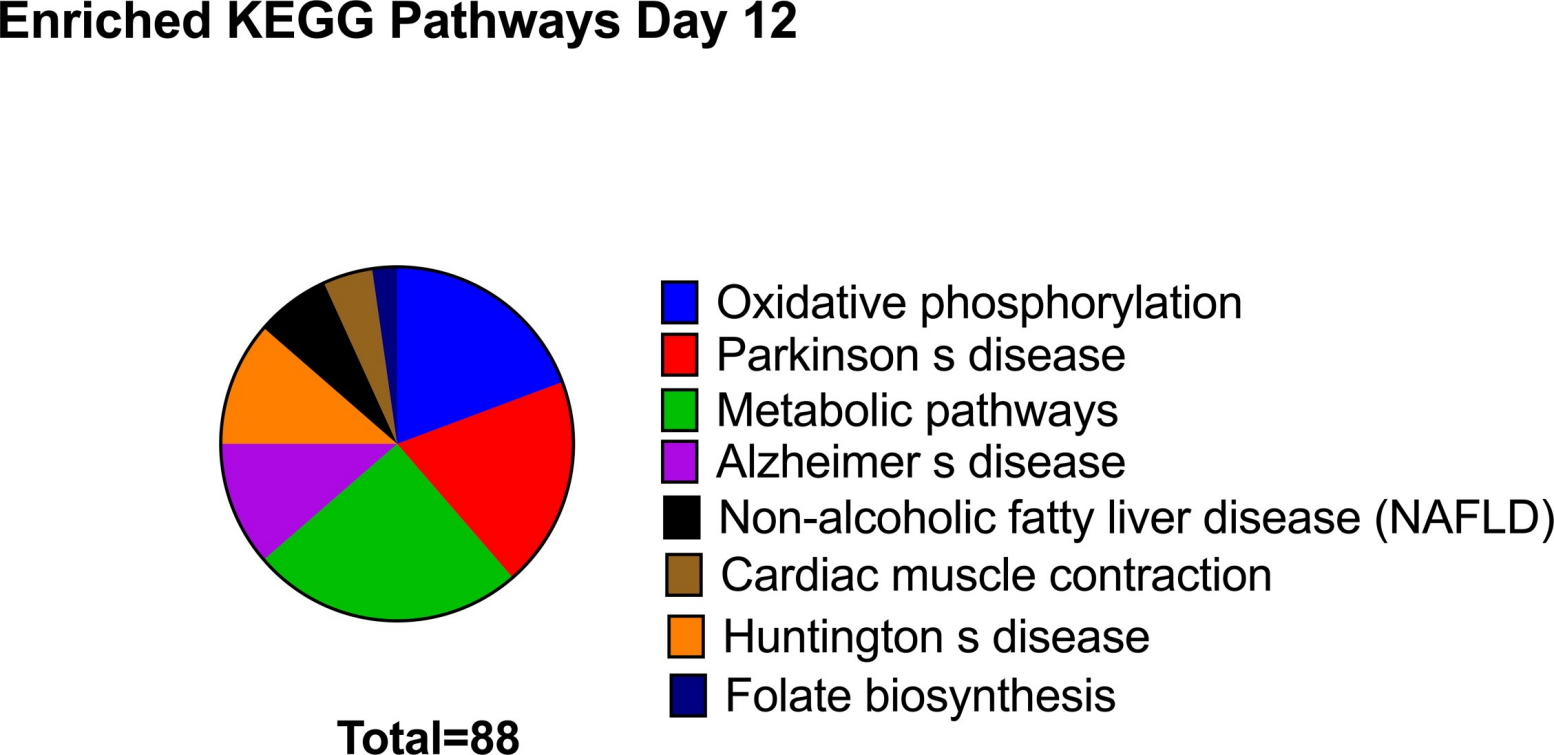
Enriched Kyoto Encyclopedia of Genes and Genomes (KEGG) pathways in transcripts downregulated in 12-Day embryos obtained with frozen thawed spermatozoa.

Of the 157 transcripts showing decreased abundance in Day-12 CRYO embryos, 60 transcripts aligned to the genome build (S6 Table). Enriched KEGG pathways, also detected in 8- and 10-day embryos, included oxidative phosphorylation, Parkinson disease, metabolic pathways, Alzheimer disease, Huntington disease, non-alcoholic fatty acid liver disease and cardiac muscle contraction. In addition a new pathway, folate biosynthesis, was enriched (Fig 9). GO terms enriched annotations (Fig 8B) were NADH dehydrogenase (ubiquinone) activity, mitochondrial respiratory chain complex I, nucleosome, DNA replication dependent nucleosome assembly, protein heterotetramerization, mitochondrion, respiratory chain, negative regulation of megakaryocyte differentiation, DNA template transcription initiation, ATP synthesis coupled electron transport, nuclear chromosome telomeric region, DNA binding, oxireductase activity, mitochondrial inner membrane, integral component of membrane, mitochondrial electron transport NADH to ubiquinone, and extracellular exosome. The complete list is given in Table 4

**Table 4:** Functional annotation chart of differentially expressed genes (downregulated) in Day-12 equine embryos obtained after AI with frozen-thawed sperm.

Category	Term	Count	PValue
UP_KEYWORDS	Membrane	22	0,035165176
GOTERM_CC_DIRECT	GO:0016021~Integral component of membrane	19	0,023827798
KEGG_PATHWAY	ecb01100:Metabolic pathways	18	8,33E-05
GOTERM_CC_DIRECT	GO:0005739~Mitochondrion	14	1,40E-06
KEGG_PATHWAY	ecb00190:Oxidative phosphorylation	13	1,69E-12
KEGG_PATHWAY	ecb05012:Parkinson's disease	13	3,57E-12
GOTERM_CC_DIRECT	GO:0070062~Extracellular exosome	13	0,047249394
UP_KEYWORDS	Chromosome	10	4,30E-12
UP_KEYWORDS	Mitochondrion	10	1,63E-10
UP_KEYWORDS	DNA-binding	10	2,09E-05
UP_KEYWORDS	Transport	10	3,60E-05
UP_KEYWORDS	Nucleus	10	6,21E-04
UP_KEYWORDS	Respiratory chain	9	7,16E-16
UP_KEYWORDS	Electron transport	9	3,97E-14
UP_KEYWORDS	Nucleosome core	9	2,19E-11
INTERPRO	IPR009072:Histone-fold	9	2,07E-10
UP_KEYWORDS	Ubiquinone	8	3,96E-16
GOTERM_MF_DIRECT	GO:0008137~NADH dehydrogenase (ubiquinone) activity	8	3,09E-12
GOTERM_CC_DIRECT	GO:0005747~Mitochondrial respiratory chain complex I	8	6,67E-11
GOTERM_MF_DIRECT	GO:0003677~DNA binding	8	5,87E-04
UP_SEQ_FEATURE	Transmembrane region	8	9,18E-04
GOTERM_CC_DIRECT	GO:0000786~Nucleosome	7	1,55E-08
UP_KEYWORDS	NAD	7	5,92E-08
KEGG_PATHWAY	ecb05322:Systemic lupus erythematosus	7	3,03E-05
UP_KEYWORDS	Oxidoreductase	7	4,97E-05
KEGG_PATHWAY	ecb05034:Alcoholism	7	2,10E-04
GOTERM_CC_DIRECT	GO:0016020~Membrane	7	0,047593153
UP_KEYWORDS	Mitochondrion inner membrane	6	2,81E-07
KEGG_PATHWAY	ecb05010:Alzheimer's disease	6	0,001939005
KEGG_PATHWAY	ecb05016:Huntington's disease	6	0,003145256
GOTERM_BP_DIRECT	GO:0006335~DNA replication-dependent nucleosome assembly	5	4,77E-07
GOTERM_BP_DIRECT	GO:0051290~Protein heterotetramerization	5	5,76E-07
INTERPRO	IPR007125:Histone core	5	2,52E-05
GOTERM_CC_DIRECT	GO:0000784~Nuclear chromosome, telomeric region	5	2,30E-04
GOTERM_CC_DIRECT	GO:0005743~Mitochondrial inner membrane	5	0,001683893
SMART	SM00417:H4	4	6,25E-07
SMART	SM00803:TAF	4	1,22E-06
GOTERM_CC_DIRECT	GO:0070469~Respiratory chain	4	2,03E-06
INTERPRO	IPR019809:Histone H4, conserved site	4	2,80E-06
INTERPRO	IPR001951:Histone H4	4	2,80E-06
GOTERM_BP_DIRECT	GO:0045653~Negative regulation of megakaryocyte differentiation	4	4,00E-06
INTERPRO	IPR004823:TATA box binding protein associated factor (TAF)	4	5,47E-06
GOTERM_BP_DIRECT	GO:0006336~DNA replication-independent nucleosome assembly	4	1,95E-05
GOTERM_BP_DIRECT	GO:0006352~DNA-templated transcription, initiation	4	2,71E-05
INTERPRO	IPR020904:Short-chain dehydrogenase/reductase, conserved site	4	1,57E-04
INTERPRO	IPR002347:Glucose/ribitol dehydrogenase	4	6,29E-04
GOTERM_BP_DIRECT	GO:0006334~Nucleosome assembly	4	8,65E-04
GOTERM_MF_DIRECT	GO:0016491~Oxidoreductase activity	4	0,001585835
INTERPRO	IPR016040:NAD(P)-binding domain	4	0,016019866
KEGG_PATHWAY	ecb04932:Non-alcoholic fatty liver disease (NAFLD)	4	0,045179763
INTERPRO	IPR001750:NADH:ubiquinone/plastoquinone oxidoreductase	3	3,19E-05
GOTERM_BP_DIRECT	GO:0042773~ATP synthesis coupled electron transport	3	5,21E-05
GOTERM_CC_DIRECT	GO:0000788~Nuclear nucleosome	3	0,004620686
KEGG_PATHWAY	ecb04978:Mineral absorption	3	0,022822957
GOTERM_CC_DIRECT	GO:0000790~Nuclear chromatin	3	0,073903618

**Fig 10:**
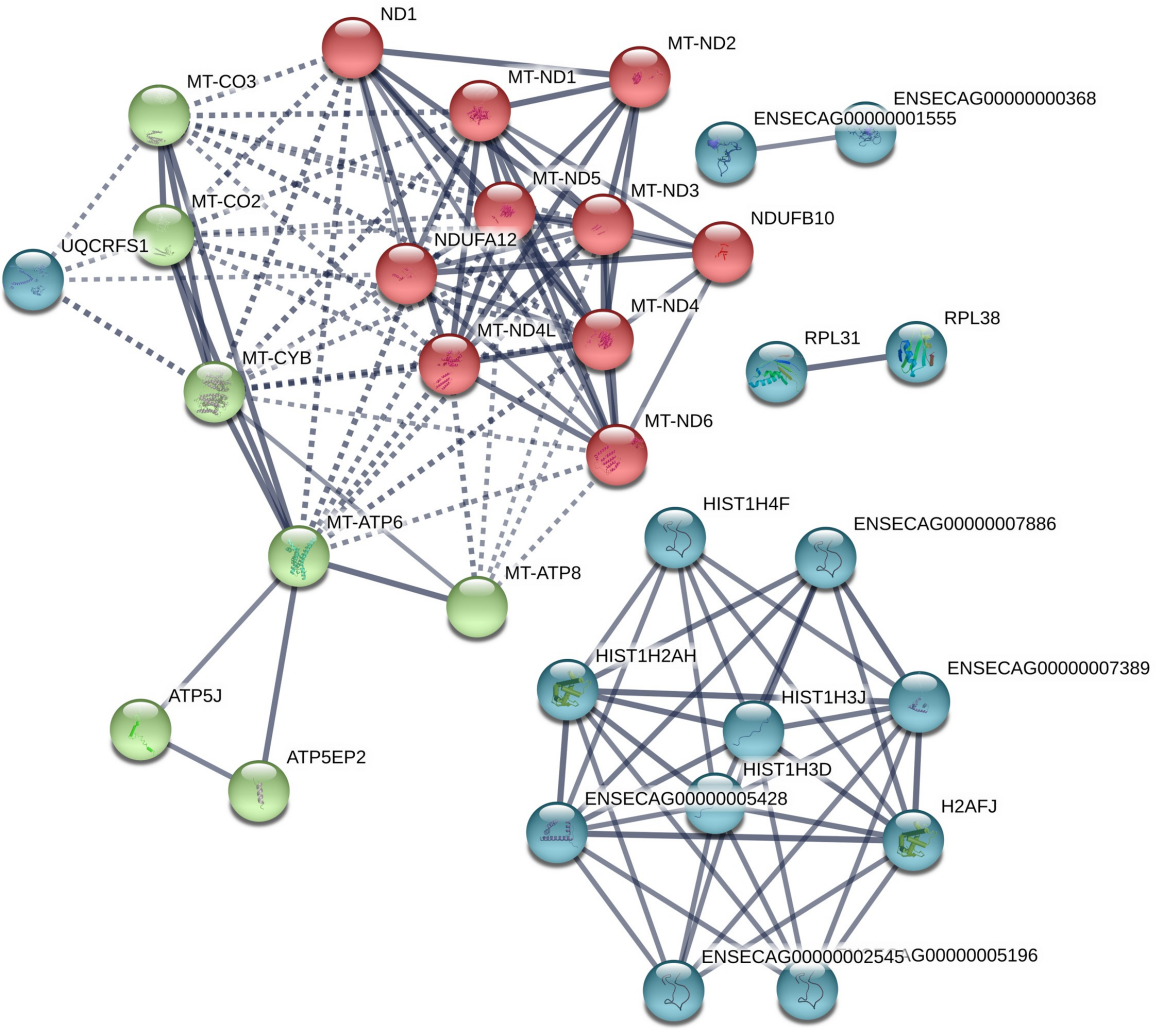
Functional networks (STRING) of transcripts downregulated in 12-Day equine embryos obtained with frozen thawed sperm (CRYO). Functional networks apply to histones and mitochondrial proteins. Controls are same-age embryos from the same mare obtained with fresh semen from the same ejaculate that was frozen and used to produce the CRYO embryos.

STRING analysis revealed functional networks with a PPI enrichment P value of < 1.0 x10^-16^ (Fig 10). Functional enrichment included the PFAM protein domains core histone H2A/H2B/H3/H4, NADH dehydrogenase, and NADH-ubiquinone/plastoquinone (complex I various chains).

### Comparison of downregulated genes with the mouse genome database

In order to explore mechanisms that may relate to reduced viability in embryos obtained using cryopreserved semen, the Mouse Genome Database [34, 35] was queried to determine whether genes downregulated in CRYO equine embryos were orthologs to mouse genes with known associations with embryo lethality.

### Day-8 embryos

In Day-8 CRYO embryos, transcripts of genes associated with the following terms were found to be of low abundance: failure of zygotic division, decreased embryo size, abnormal embryo size, embryonic growth arrest, embryonic growth retardation, embryonic lethality before implantation-complete penetrance, embryonic lethality between implantation and somite formation-complete penetrance, embryonic lethality between somite formation and embryo turning-complete penetrance, embryonic lethality prior to tooth bud stage, abnormal embryonic tissue morphology, abnormal extraembryonic tissue morphology, delayed allantois development, perinatal lethality incomplete penetrance, prenatal lethality-complete penetrance, preweaning lethality-complete penetrance, abnormal male germ cell apoptosis, abnormal spermatogenesis, azoospermia, male infertility, and female infertility.

### Day 10 embryos

In Day-10 CRYO embryos, the following gene associations cited above for Day-8 embryos were found: decreased embryo size, abnormal embryo size, failure of zygotic cell division, embryonic lethality between implantation and somite formation, embryonic lethality between implantation and somite formation-complete penetrance, embryonic lethality prior to tooth bud stage, prenatal lethality-complete penetrance, perinatal lethality-incomplete penetrance, preweaning lethality, preweaning lethality-complete penetrance, and abnormal spermatogenesis.

In addition, the following associations were found: abnormal blastocyst morphology, absent blastocele, abnormal inner cell mass morphology, absent inner cell mass proliferation, empty decidua capsularis, embryonic growth retardation, failure of blastocyst to hatch from the zona pellucida, abnormal preimplantation embryo development, failure to gastrulate, embryonic lethality prior to embryogenesis, failure of embryo implantation, abnormal decidua basalis morphology, abnormal extraembryonic endoderm formation, prenatal lethality prior to heart atrial septation, decreased fetal size, preweaning lethality incomplete penetrance, abnormal gametogenesis, abnormal spermatid morphology, abnormal vas deferens morphology, decreased mature ovarian follicle number, reduced female fertility and small ovary.

### Day 12 embryos

In 12-day CRYO embryos the following gene associations cited above were found: abnormal embryo size, decreased embryo size, prenatal lethality prior to heart atrial septation, embryonic lethality prior to tooth bud stage, preweaning lethality-complete penetrance, male infertility, female infertility, and small ovary.

In addition, the following associations were found: incomplete embryo turning, embryonic lethality prior to organogenesis, embryonic lethality during organogenesis-complete penetrance, decreased FSH level, small seminal vesicle, small seminiferous tubules, small testis, absent mature ovarian follicles, abnormal ovulation, abnormal corpus luteum morphology, uterus hypoplasia, and vaginal atresia.

## Discussion

Here we report, for the first time, evidence that procedures performed during handling of sperm, such as freezing and thawing, have a significant impact on critical aspects of the early embryo transcriptome. The equine model used in our study has a number of advantages, including a long pre-attachment embryonic period in which the embryo remains spherical, which facilitates embryo collection, and the possibility of repeated embryo collections from the same animals over successive estrus cycles. Additionally, the stallion serves as an excellent model for the human male, as stallions are typically not selected for sperm quality nor the ability of semen to be cryopreserved, in contrast to males in production species, such as the bull. Moreover, since many stallions reach advanced age, the horse can be used as a model to study the impact of paternal age on embryo quality.

Our study, focused on three embryo ages (8, 10 and 12 days post ovulation), revealed a significant impact of sperm cryopreservation on the transcriptome of the resulting embryo. Importantly, transcripts with decreased abundance reflected genes related to DNA replication and assembly, and oxidative phosphorylation. Exploration of differentially-expressed genes at the molecular and cellular level revealed alterations in important functions including ATP synthesis, regulation of transcription, nucleosome assembly, chromatin silencing, protein synthesis, and redox regulation. Alterations in these genes help to explain the reduced fertility observed with cryopreserved sperm attributable to increased early embryo mortality [11, 12].

The pre-implantation period is a period of rapid embryo growth, requiring a ready supply of ATP. The equine embryo appears to have a significant capacity for glycolysis, but also uses oxidative phosphorylation [36]. The KEGG pathways analysis of downregulated genes revealed enriched annotations for oxidative phosphorylation, pyruvate metabolism, glycolysis, and the TCA cycle, suggesting compromised energy metabolism in CRYO embryos. A similar picture was observed in Day-10 and Day-12 embryos, with the pathways for oxidative phosphorylation, metabolic pathways, and non alcoholic fatty liver disease significantly over-represented in transcripts with reduced abundance of all CRYO embryos obtained.

When we evaluated low-abundance equine transcripts for their mouse orthologs, we found that many of the genes downregulated in CRYO embryos have knockout database annotation terms related to reduced embryonic viability. This finding opens the possibility that not only genes related to the metabolism and thus growth of embryos, but also genes directly related to embryo organogenesis, embryo survival, and offspring health are affected by the use of cryopreserved sperm, and thus these genes warrant further investigation.

While the mechanisms behind the effects reported here are as yet unclear, a major factor may be the well-documented oxidative damage that the genome and epigenome experiences during cryopreservation and thawing [11–14]. Cryopreservation is a major cause of oxidative stress [37] and lipid peroxidation in stallion spermatozoa [10, 17, 38, 39]. Lipid peroxidation in spermatozoa surviving cryopreservation [37] is associated with increased levels of 4-hydroxinonenal (4-HNE) [17]. This compound is able to interact with DNA to form adducts that have been related directly to increased rates of mutation in important cell-cycle regulators [40, 41]. The production of 4-HNE during cryopreservation of stallion spermatozoa is well documented [10, 17, 39], and it is possible that significant amounts of 4-HNE and other toxic lipid aldehydes are incorporated to the oocyte, potentially causing alterations in embryo development. In addition to DNA damage, 4-HNE can alkylate the sperm centrioles, and in horses, as in humans, paternal centrioles are inherited by the embryos. Damaged centrioles may cause disrupted cytoskeletal protein organization during early cleavage [42].

Supporting this line of reasoning, recent reports have linked abnormal early cleavage events and changes in embryo transcript abundance to fertilization with spermatozoa showing oxidative stress. Macaque embryos obtained after fertilization with ROS-treated sperm showed significantly lower rates of development to the four- and eight-cell stages, and changes in transcript abundance for genes related to actin cytoskeleton organization, cell junction assembly and cell adhesion [43]. Although seen at a much later stage of development, in our study we also found that genes for cytoskeleton components tubulin alpha 1 a, tubulin beta 2 class II a and actin, cytoplasmic 1, N-terminally processed were downregulated in 8-day CRYO embryos.

Cryopreservation may also directly affect the epigenome of the paternal DNA; recent studies have shown that cryopreservation increases the level of DNA methylation in equine sperm [12] and the expression of genes important to intracellular regulation of epigenetic status [16]. Notably, we also found significant reduction in abundance of transcripts for histones in CRYO embryos.

The finding that many differentially regulated genes in CRYO embryos are orthologs of mouse genes that have knockout database annotation terms related to reduced embryonic viability provides further evidence linking cryopreserved sperm to reduced embryonic viability. These annotations consistently appeared on analysis of low-abundance transcripts in all CRYO embryos, and included genes related to embryonic growth retardation and embryo lethality. Interestingly, annotations related to male and female infertility were also present; this warrants further investigation on the effect of sperm origin on the fertility of resulting offspring.

In summary, the present study provides for the first time transcriptomic analysis of equine embryos in relation to the handling of semen used for their production, however we acknowledge the preliminary and descriptive nature of this report but our data provide strong evidence that cryopreservation of sperm exerts a profound impact on the transcriptome of early embryos. Our findings may stimulate new lines of research to improve this biotechnology in humans and animals.

## Supporting information

S1 Table Transcripts upregulated in 8-Day embryos obtained with frozen-thawed spermatozoa with respect to controls obtained with fresh semen. (XLSX)

S2 Table Transcripts downregulated in 8-Day embryos obtained with frozen-thawed spermatozoa. (XLSX)

S3 Table Network analysis of transcripts downregulated in 8-Day embryos obtained with frozen-thawed spermatozoa. List of transcripts in each cluster obtained after STRING analysis; genes in each cluster are presented and colors for each cluster are given. Network is presented in Fig 4.
(XLSX)

S4 Table Transcripts upregulated in 10-Day embryos obtained with frozen-thawed spermatozoa. (XLSX)

S5 Table Transcripts upregulated in 12-day embryos obtained with frozen-thawed spermatozoa. (XLSX)

S6 Table Transcripts downregulated in 12-Day embryos obtained with frozen-thawed spermatozoa. (XLSX)
